# Age-Related Features of Neuroinflammation: Hidden Association of Neuronal Damage with Activation of Natural Killers in Patients with Ischemic Stroke

**DOI:** 10.3390/ijms262311452

**Published:** 2025-11-26

**Authors:** Matvey Vadyukhin, Tatiana Demura, Eugenia Kogan, Vladimir Shchekin, Petr Shegai, Andrey Kaprin, Grigory Demyashkin

**Affiliations:** 1 Department of Digital Oncomorphology, National Medical Research Centre of Radiology, 2nd Botkinsky Pass., 3, 125284 Moscow, Russia; dr.shchekin@mail.ru (V.S.);; 2 Institute of Translational Medicine and Biotechnology, I.M. Sechenov First Moscow State Medical University (Sechenov University), Trubetskaya st., 8/2, 119048 Moscow, Russia; dr.dga@mail.ru; 3 Institute of Clinical Morphology and Digital Pathology, I.M. Sechenov First Moscow State Medical Univesity (Sechenov University), Trubetskaya st., 8/2, 119048 Moscow, Russia; 4 Research and Educational Resource Center for Immunophenotyping, Digital Spatial Profiling and Ultrastructural Analysis Innovative Technologies, Peoples’ Friendship University of Russia (RUDN University), Miklukho-Maklaya st., 6, 117198 Moscow, Russia; 5 Department of Urology and Operative Nephrology, Peoples’ Friendship University of Russia (RUDN University), Miklukho-Maklaya st., 6, 117198 Moscow, Russia

**Keywords:** ischemic stroke, neuroinflammation, NK cells, NKT cells, aging

## Abstract

Ischemic stroke remains a leading cause of death and disability worldwide, with neuroinflammation playing a central role in its pathogenesis. This study aimed to investigate age-related differences in neuroinflammatory responses in the human cerebral cortex following ischemic stroke. Using autopsy-derived brain tissue from 184 patients histological, histochemical, multiplex immunofluorescence, ELISA, and qRT-PCR analyses were conducted to assess neuronal damage, immune cell infiltration and cytokine expression. Morphological examination revealed pannecrosis in infarct cores and moderate inflammatory infiltration in penumbral regions. Multiplex immunofluorescence demonstrated active migration of NK (CD45^+^CD56^+^CD3^−^) and NKT (CD45^+^CD56^+^CD3^+^) cells in young patients, whereas elderly individuals showed a predominance of T lymphocytes (CD45^+^CD56^−^CD3^+^) and a decline in NK/NKT activity. Molecular assays indicated elevated NKG2D receptor expression and higher IFN-γ and proinflammatory cytokine (TNF-α, IL-1β, IL-6) levels in young patients, contrasting with dysregulated cytokine balance and reduced NK cytotoxicity in the elderly. These findings highlight distinct age-dependent immune mechanisms underlying ischemic injury, demonstrating that NK and NKT cells play a crucial role in early neuroinflammation, while aging shifts the balance toward adaptive immune dominance. The study underscores the need to consider patient age when designing neuroprotective and anti-inflammatory therapeutic strategies for ischemic stroke.

## 1. Introduction

Ischemic stroke remains one of the leading causes of mortality and long-term disability worldwide. According to the Global Burden of Disease (GBD) study, in 2021 there were 7.8 million (95% CI: 6.7–8.9 million) new cases of ischemic stroke globally, with an age-standardized incidence rate of 92.4 per 100,000 population; between 1990 and 2021, the overall number of cases increased by 87.7% [1]. Among Eastern European countries, the Russian Federation occupies one of the top positions in terms of disease incidence: in 2021, 490,197 cases of ischemic stroke were reported, corresponding to an age-standardized rate of 142.6 per 100,000 population [2].

Among the diverse inflammatory cell populations involved in post-stroke pathology, the role of natural killer (NK) cells in the initiation and maintenance of neuroinflammation appears particularly significant [3]. Several studies have demonstrated early infiltration of both the infarct core and penumbra by NK cells within the first hours following stroke onset, with their activity peaking during the first three days, as supported by recent experimental findings [4].

Particular attention has been directed toward the molecular mechanisms underlying NK cell activation, migration, and cytotoxicity during ischemic stroke, as their precise contribution within the penumbral zone remains insufficiently characterized. It has been proposed that NK cells mediate nonspecific immune reactions in ischemic regions and that their released effectors—such as perforin, granzymes, and interferon-gamma (IFN-γ)—may exacerbate neuronal damage in areas that are potentially reversible during the acute phase of stroke, thereby expanding the infarct core [5,6].

Recent reports have described interactions between the NKG2D receptor, a type II transmembrane immunoreceptor expressed on the surface of NK cells, and its ligands (MICA/B, ULBP1–6), which are upregulated on neurons and glial cells in response to hypoxia in ischemic stroke models using C57BL/6N mice [7]. This concept is further supported by studies in which NKG2D (*KLRK1*) gene silencing attenuated neuroinflammation in neonatal murine models of hypoxic–ischemic brain damage (HIBD). However, the structure and diversity of NKG2D ligands differ substantially between humans and mice: in murine systems, the receptor exists in two isoforms (NKG2D-L and NKG2D-S) and is activated by Rae-1, H60, and MULT-1, whereas in humans, the principal ligands are the stress-induced proteins MICA/B and ULBP family members [8]. These interspecies distinctions may underlie fundamental differences in the molecular mechanisms regulating NK cell migration and activation following ischemia, emphasizing the need for studies utilizing human brain tissue.

The downstream consequences of NKG2D engagement include NK cell migration to ischemic foci and activation of the intracellular phosphoinositide 3-kinase/Akt kinase (PI3K/AKT) signaling pathway, which promotes the transcription of proinflammatory cytokines [9]. This cascade not only enhances the cytotoxic functions of NK cells themselves but also amplifies the activity of other inflammatory effectors within the ischemic microenvironment [10].

Despite the emerging evidence, the precise contribution of NK cells to post-ischemic neuroinflammation remains poorly understood and warrants comprehensive investigation. Moreover, it is not yet established whether patient age modulates the magnitude or dynamics of nonspecific immune responses after stroke. Understanding the interactions between NK cells and cortical neurons is of particular importance, as modulation of these mechanisms may reduce the cytotoxic potential of immune cells within the penumbra and prevent expansion of the infarct core. This concept opens a promising avenue for the development of personalized therapeutic strategies based on age-dependent features of neuroinflammation.

While numerous studies have described the general role of immune mechanisms in the pathogenesis of ischemic stroke, far less attention has been given to how these processes differ across age groups. Most existing research focuses on either experimental models or individual components of the immune response, without integrating morphological, immunophenotypic, and molecular changes in human brain tissue. Therefore, the present study does not merely confirm the involvement of neuroinflammation in ischemic injury, but places a specific emphasis on age-related differences in innate and adaptive immune responses. By comparing NK, NKT, and T lymphocyte dynamics along with oxidative stress and cytokine profiles in young, middle-aged, and elderly patients, this work seeks to uncover novel age-dependent mechanisms that may explain the variability in clinical outcomes and therapeutic efficacy.

The aim of this study was to conduct a comprehensive assessment of age-related features of neuroinflammation in the cerebral cortex of patients with ischemic stroke. Specifically, we sought to elucidate the relationship between neuronal damage, NK and NKT cell migration and T-cell infiltration using multiplex immunohistochemical analysis within the penumbra during the early stages of ischemia.

## 2. Results

A total of 154 patients were divided into young (n = 38), middle (n = 51), and elderly (n = 65) aged groups: mean ages were 34.8 ± 4.2, 51.1 ± 4.8, and 69.3 ± 5.4 years, respectively (*p* < 0.0001), with no significant sex differences (Table 1). According to ICD-10, most cases were infarcts in the middle cerebral artery location, while diagnoses coded as I67.8 increased with age (*p* < 0.0001). By SSS-TOAST, cardioembolic genesis predominated in the young, whereas atherosclerotic was more frequent in the elderly, though not statistically significant. Risk factors showed a marked rise in hypodynamia and hyperlipidemia with age (both *p* < 0.0001), while smoking, obesity, and hypertension were comparable across groups. Mortality patterns differed: young patients most often died on day 3 (34.2%), middle-aged on days 1–2 (47.0% and 23.5%). The elderly group showed a more even distribution of mortality across the first week; however, significant differences were observed between the young and elderly groups on Day 1 (*p* = 0.007), and between the middle-aged and elderly groups on Day 3 (*p* = 0.035). No significant differences were found between the other age group comparisons on these days. On Days 4–7 the number of deaths in each age group was low; therefore, these data are presented descriptively and were not used for formal statistical comparison, except where sufficient counts allowed.

**Figure 1:**
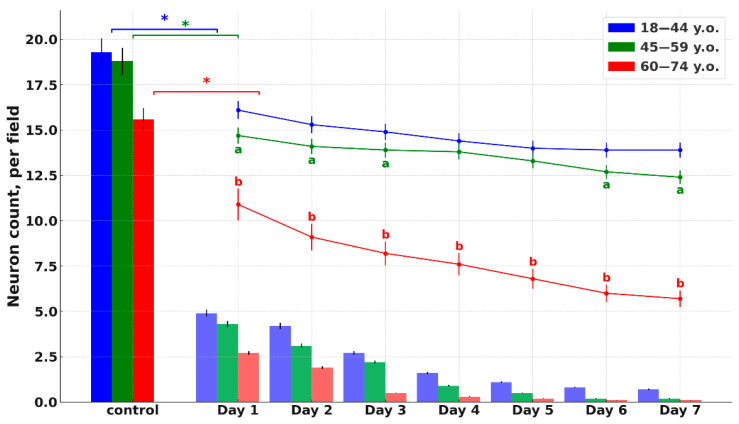
Results of morphometric analysis of the number of neurons (in the visual field) in the control and in the group of ischemic stroke patients of young (blue), middle (green) and elderly (red) age, graph. The columns indicate the number of neurons in the infarct zone, the colored curves indicate the number of neurons in the penumbra. Statistically significant differences: *—“control” vs. “Day 1”, ^a^—middle vs. young, ^b^—elderly vs. young; *p* ≤ 0.05.

**Figure 2:**
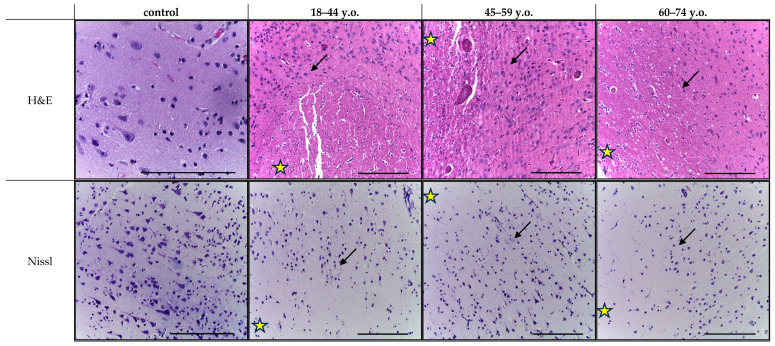
Frontal lobe of the cerebral cortex in the control (middle-aged patient) and ischemic stroke group in the young (18–44 y.o.), middle (45–59 y.o.) and elderly (60–74 y.o.) age subgroups: hematoxylin and eosin staining (on the left); Nissl staining (on the right), magn. ×200. The micrographs visualize: the infarct zone (yellow star), the penumbra zone (black arrow). Scale bar—50 μm.

Histological analysis of cortical fragments in the ischemic stroke group revealed areas of pannecrosis with reduced neuronal density, eosinophilic perikaryal cytoplasm, and nuclear pyknosis (Figure 1). Infarct zones showed microcirculatory disturbances, including venous congestion, perivascular edema, erythrocyte aggregation, and stasis in small vessels. In the penumbra, structural alterations were less pronounced and were accompanied by sparse perivascular lymphocytic and occasional polymorphonuclear cell infiltration, as observed in H&E-stained sections (Figure 2). Age comparison indicated slightly weaker ischemic alterations in older patients, along with cortical thinning and small demyelination foci in both controls and stroke cases. Nissl staining confirmed marked neuronal loss within the infarct core and partial neuronal preservation in the penumbra. Across age groups, controls and stroke patients exhibited fewer neurons, thinner axons, and weaker perikaryal staining in the elderly compared to younger individuals (Figure 1 and Figure 2). Similar neuronal thinning and reduced Nissl staining were also observed in elderly controls, indicating that these changes reflect age-related cortical alterations rather than consequences of stroke alone.

Histological analysis revealed no inflammatory cell infiltration in the control group at any age. Immunohistochemical analysis with antibodies to CD56, CD45, and CD3 revealed isolated positive NK cells (CD45^+^CD56^+^CD3^−^, green/orange signal) and T lymphocytes (CD45^+^CD56^−^CD3^+^, yellow/orange signal) only in the lumen of blood vessels. Some cells in the brain tissue were also positive for CD45, which may be normal for obligate cells of macrophage origin (microglia) with characteristic morphology. Based on these findings, particular attention was paid to comparing immune cells migration and distribution between different age groups of patients with ischemic stroke during the multiplex immunohistochemical study. Additionally, immunophenotyping of the detected immune cells was performed using antibodies to the listed markers.

**Figure 3:**
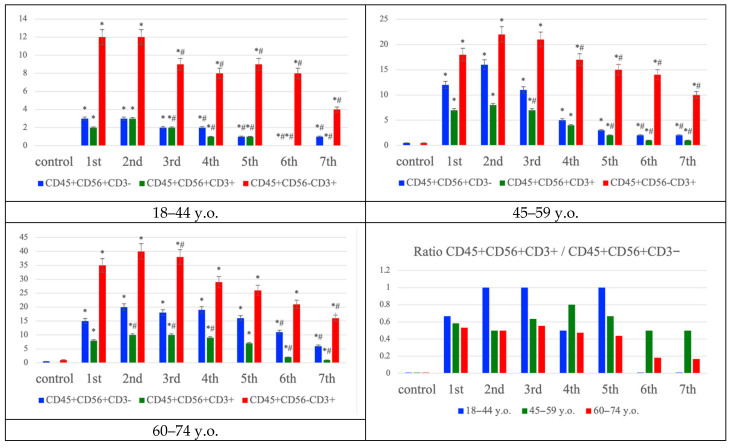
Distribution of NK (CD45^+^CD56^+^CD3^−^ phenotype), NK T cells (CD45^+^CD56^+^CD3^+^ phenotype), and T lymphocytes (CD45^+^CD56^−^CD3^+^ phenotype) in the frontal lobe of the cerebral cortex in the control group and in patients with ischemic stroke at young (18–44 y.o.), middle (45–59 y.o.), and elderly (60–74 y.o.) ages at the first week, graph. Statistically significant differences: *—“control” vs. “Day 1”, ^#^—compared to “Day 1”; *p* ≤ 0.05.

**Figure 4:**
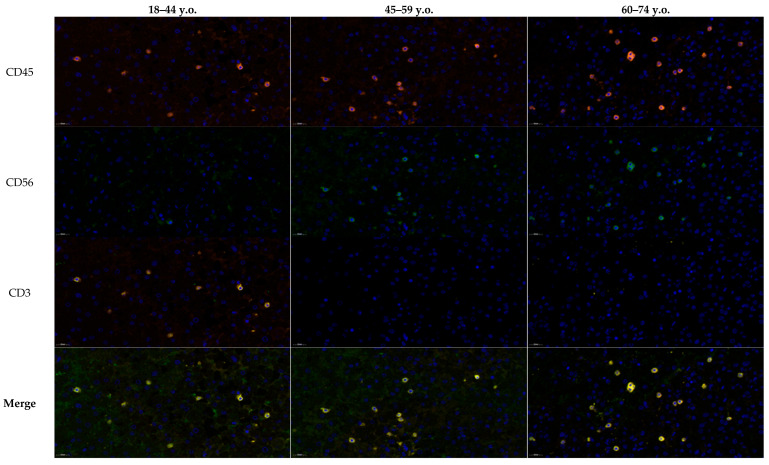
Frontal cortex of the brain of young (18–44 y.o.), middle (45–59 y.o.) and elderly (60–74 y.o.) aged patients with ischemic stroke on the first week. Multiplex microscopy with antibodies to immune cell markers CD45 (orange glow, Opal 647), CD56 (green glow, Opal 488) and CD3 (yellow glow, Opal 540). NK cells react positively to CD45 and CD56 (CD45^+^CD56^+^CD3^−^ phenotype); NK T cells react positively to CD45, CD56 and CD3 (CD45^+^CD56^+^CD3^+^ phenotype); T lymphocytes react positively to CD45 and CD3 (CD45^+^CD56^−^CD3^+^ phenotype); DAPI—blue signaling.

Multiplex immunofluorescence analysis of the penumbra demonstrated age-dependent differences in the distribution of immune cell phenotypes (Figure 3). In younger patients (18–44 years), NK cells (CD45^+^CD56^+^CD3^−^, green/orange signal) and T lymphocytes (CD45^+^CD56^−^CD3^+^, yellow/orange signal) were both detectable from the first day, with NK T cells (CD45^+^CD56^+^CD3^+^) comprising a smaller fraction. In middle-aged patients (45–59 years), the number of NK and T cells increased, accompanied by a moderate rise in NK T cells. In the elderly group (60–74 years), T lymphocytes dominated the infiltrate, while the relative proportion of NK T cells to NK cells progressively declined over time, as confirmed by ratio analysis. These findings suggest a shift from a more balanced NK–T cell response in younger individuals toward T lymphocyte predominance and reduced NK T cell contribution with aging, reflecting age-related alterations in post-ischemic immune regulation (Figure 4). While Figure 3 summarizes the quantitative distribution of NK, NKT and T cells, Figure 4 complements these findings by demonstrating typical cellular localization and marker expression patterns in situ.

**Figure 5:**
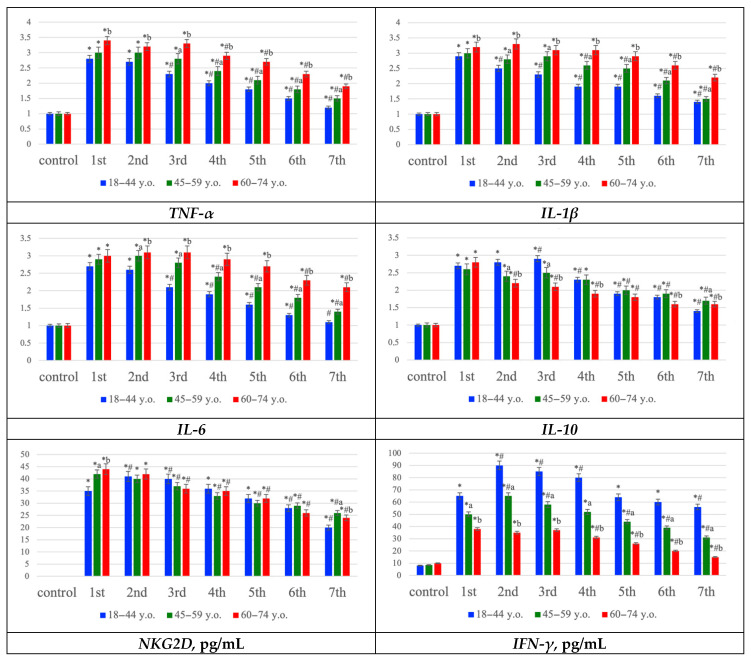
Concentrations of NK cell receptor NKG2D (pg/mL), and interferon-γ (pg/mL), as well as the relative mRNA expression levels of the cytokine genes TNF-α, IL-1β, IL-6, and IL-10 in the frontal lobe tissue homogenate of the cerebral cortex of the control group and in patients with ischemic stroke at young (18–44 y.o.), middle (45–59 y.o.), and elderly (60–74 y.o.) ages at the first week, graph. Statistically significant differences: *—“control” vs. “Day 1”, ^#^—compared to “Day 1”, ^a^—middle vs. young, ^b^—elderly vs. young; *p* ≤ 0.05.

Gene expression profiling showed upregulation of proinflammatory mediators (TNF-α, IL-1β, IL-6) and the anti-inflammatory cytokine IL-10, most prominently during the first three days post-stroke, with expression levels generally higher in older patients. In addition, NKG2D and IFN-γ concentrations increased early, particularly in young patients, but declined more rapidly in the elderly, suggesting reduced persistence of NK- and T-cell–mediated immune activity with age. Collectively, these data indicate that ischemic stroke is accompanied by strong oxidative stress and inflammatory activation across all age groups, but the elderly exhibit more pronounced oxidative damage, prolonged proinflammatory signaling, and a diminished compensatory immune response (Figure 5).

## 3. Discussion

This study presents a comprehensive analysis of morphological, molecular, and immunological alterations in the cerebral cortex of patients with ischemic stroke across different age groups. The obtained data reveal both universal pathogenic mechanisms common to all ages and distinct age-related differences that may contribute to the heterogeneity of clinical manifestations [11,12,13]. Particularly these findings may explain the variable efficacy of acute stroke therapy and subsequent neurorehabilitation observed among patients of different age groups [14].

Histological examination revealed classical features of ischemic stroke, including the formation of infarct core and penumbra zones [15]. In all groups, the infarct core was characterized by pannecrosis with markedly reduced neuronal density, eosinophilic perikaryal cytoplasm, and nuclear pyknosis [16]. These alterations were accompanied by microcirculatory disturbances, such as venous congestion, perivascular edema, erythrocyte aggregation, and blood stasis within capillaries and arterioles. In contrast, the penumbra demonstrated milder structural changes combined with moderate lymphocytic and polymorphonuclear infiltration based on H&E analysis results. Comparative analysis among age groups revealed that in elderly patients, these morphological changes were more pronounced, and the infarct and penumbral zones were broader, likely reflecting reduced tissue reactivity and diminished compensatory mechanisms of the aging brain, including collateral circulation [17,18]. Cortical thinning and small demyelinating foci observed in control samples from older individuals further support the presence of background age-associated degenerative processes that may decrease neural adaptability and increase cortical vulnerability to ischemic injury [19,20]. This assumption is consistent with clinical observations in patients diagnosed with ischemic stroke in the context of Alzheimer’s disease and other forms of dementia [21].

It should be emphasized that the reduced neuronal density, thinner axons and weaker perikaryal staining observed in elderly patients were also present in the age-matched control group. Therefore, these alterations most likely reflect age-related cortical remodeling rather than stroke-induced damage per se. We included these data to delineate the baseline structural differences between age groups, as such age-associated neuronal vulnerability and pre-existing degeneration may modulate the extent of ischemic injury and partially explain the wider infarct and penumbra zones observed in older individuals.

A particular focus of this study was the age-dependent redistribution of lymphocyte phenotypes within the inflammatory infiltrate during ischemic stroke. In younger patients, we observed a more balanced and coordinated immune response, whereas in elderly individuals, T lymphocytes predominated and cytokine imbalance was evident. Moreover, our data indicate that with aging, the contribution of NK and NKT cells to the early immune response declines, reflecting age-related immunoregulatory alterations that may partially explain differences in clinical stroke outcomes [22]. Dysregulation of inflammatory signaling—characterized by an imbalance in cytokine expression—and the predominance of T cells in older patients likely promote nonspecific cytotoxic neuronal death within the penumbra, contributing to a more prolonged expansion of the infarct core [23]. In contrast, younger individuals exhibited a more balanced profile of pro- and anti-inflammatory cytokines and a lower overall degree of immune cell infiltration, even during the early post-stroke period.

Immunohistochemical analysis revealed clear age-related distinctions in the cellular composition of the inflammatory infiltrate. In young patients, both NK cells and T lymphocytes were detected within the infarct core and penumbra from the first day after stroke onset, with a moderate proportion of NKT cells. In middle-aged patients, infiltration of both NK and T cells increased, accompanied by a mild expansion of the NKT population. In elderly individuals, however, the infiltrate shifted toward a predominance of T lymphocytes, with a concomitant decline in the relative proportion of NKT cells and the NKT/NK ratio, suggesting impaired regulation and reduced functional activity of these populations [24]. Notably, while NK and NKT cells are primarily cytotoxic, a small subset may exhibit protective functions by stimulating the production of anti-inflammatory cytokines [25]. Collectively, these findings indicate an age-dependent decline in the cytotoxic potential of innate immune cells and dysregulation of inflammatory control within the penumbra. The early accumulation of NK cells in ischemic regions observed in our study may reflect their role as initiators of the inflammatory response, triggering the expression of both proinflammatory (TNF-α, IL-1, IL-6) and anti-inflammatory (IL-10) cytokines. This dynamic likely promotes the recruitment of additional immune cells and the formation of a complex microenvironment consisting of NK and T lymphocytes. Concurrently, these processes may activate intracellular signaling cascades regulating survival and inflammatory responses, including activation of phosphoinositide 3-kinase (PI3K), mitogen-activated protein kinase (MAPK), and nuclear factor-κB pathways and its downstream effectors [26].

According to the literature, elderly patients are presumed to exhibit an elevated oxidative status accompanied by reduced antioxidant defense activity [27,28]. It is possible that increased concentrations of free radicals (^1^O_2_, O_2_^−^, OH·, H_2_O_2_, NO·, ONOO^−^) in older individuals, compared with younger adults, may contribute to heightened neuronal vulnerability and cell death, as well as to the enhancement and dysregulation of proinflammatory cytokine synthesis (TNF-α, IL-1, IL-6) and the recruitment and migration of immune cells, including NK, NKT, and T lymphocytes [29,30]. However, this potential relationship remains to be clarified in future studies.

Gene expression profiling revealed an upregulation of proinflammatory cytokines (TNF-α, IL-1β, IL-6) and the anti-inflammatory cytokine IL-10, particularly during the first three days post-stroke. In younger patients, IL-10 levels remained more stable, whereas elderly individuals showed a predominant increase in proinflammatory mediators. These results suggest activation of both damaging and compensatory mechanisms in the young, while aging is associated with a proinflammatory shift and loss of regulatory balance [31]. On a molecular level, enhanced NF-κB and JAK/STAT signaling likely drives proinflammatory cytokine transcription, whereas diminished PI3K/AKT/mTOR activity in older patients may account for weakened antiapoptotic and anti-inflammatory responses [26].

Notably, the distribution of time to death differed between age groups, with middle-aged patients showing a higher concentration of deaths on Days 1–2, whereas young patients peaked on Day 3 (Table 1). These patterns align with the distinct immuno-inflammatory trajectories we observed. The early mortality in middle-aged patients is consistent with hyperacute complications of large ischemic insults—such as malignant edema, herniation, or cardiac instability—occurring before the full evolution of secondary neuroinflammation. In contrast, the Day 3 peak among young patients temporally coincides with the window of secondary injury, when lipid peroxidation (MDA) and proinflammatory mediators (TNF-α, IL-1β, IL-6) reached their maxima (Figure 5). In this cohort, we also detected an early surge of NK/NKT activity (CD45^+^CD56^+^ with/without CD3 and elevated NKG2D/IFN-γ), which may initially sustain perfusion and delay catastrophic deterioration, but subsequently amplify penumbral injury via IFN-γ–driven cytokine cascades and oxidative stress. Taken together, these observations suggest that hyperacute death in middle age may be dominated by primary injury dynamics, whereas delayed death in the young more closely reflects secondary, immune-mediated injury progression. Although our study was not designed for formal survival modeling, these hypothesis-generating data argue for age-tailored therapeutic timing—prioritizing ultra-early edema control in middle-aged patients and aggressive modulation of innate immune and redox pathways within the first 48–72 h in younger patients.

Additional evidence for the involvement of innate immune cells—specifically NK and NKT populations—was obtained by evaluating NKG2D receptor expression and IFN-γ levels, both of which increased significantly in all groups during the early stages of ischemia. However, their concentrations were highest and persisted longer in younger patients, consistent with active migration of these cells from the vasculature into cortical tissue [32,33]. In elderly patients, both parameters rose less sharply and declined more rapidly, indicating reduced migration (reflected by lower NKG2D expression) and diminished cytotoxic activity (reflected by decreased IFN-γ levels) of NK cells [9]. Thus, in younger individuals, NK cells appear to play a central role in the initiation of neuroinflammation, whereas in older patients, T lymphocyte activity becomes dominant. Some studies suggest that stressed neurons and glial cells may induce early recruitment of NK and NKT cells through upregulation of NKG2D ligands (MICA/B and ULBP1–6) [34,35,36], though this hypothesis requires further molecular validation in human tissue.

**Figure 6:**
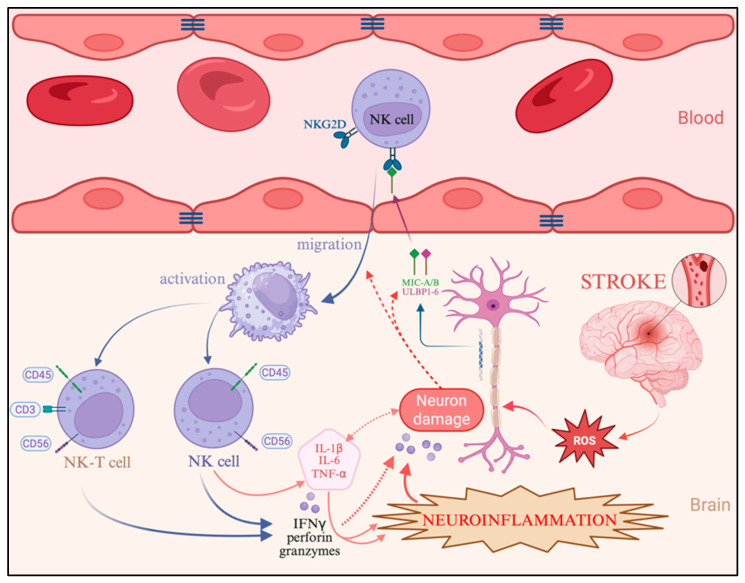
Schematic representation of the interplay between neuronal signaling, NK cell activation, and the initiation of neuroinflammation in ischemic stroke. ROS—reactive oxygen species; NK—natural killer cells; NKG2D—NKG2-D type II integral membrane receptor; MICA—MHC class I polypeptide–related sequence A; ULBP3—UL16-binding protein 3; IFN-γ—interferon gamma. Created in BioRender.com, access on 19 September 2025.

The described cellular and molecular alterations occurring in the brain during ischemic stroke are summarized schematically in Figure 6. Importantly, beyond confirming established ischemic injury mechanisms, our results highlight that age is a decisive factor shaping the neuroinflammatory response. Unlike previous studies that predominantly describe NK cell activation or cytokine production in general terms, we demonstrate that these processes follow distinct trajectories in different age groups. Younger patients show a rapid, NK/NKT-driven innate immune response with balanced cytokine regulation, whereas elderly individuals exhibit delayed but intensified T cell–mediated inflammation accompanied by oxidative dysregulation. This age-dependent divergence represents the key novel contribution of this study and suggests that therapeutic strategies should be tailored according to immunological age rather than chronological age alone.

Thus, despite the pronounced development of neuroinflammation, there are fundamental age-dependent differences in the cellular composition of the infiltrate. NK and NKT lymphocytes in young patients as well as T cells in elderly group exhibit cytotoxic activity and may also express proinflammatory cytokines, thereby amplifying the inflammatory response and contributing to neuronal death within the penumbra [10,37]. To further probe this signal, future studies should integrate time-resolved sampling with survival analyses (e.g., Cox models) that adjust for stroke subtype, vascular territory, and comorbidity burden, testing whether NK/NKT signatures (NKG2D, IFN-γ) and oxidative markers (MDA, SOD-1) independently associate with time to death across ages. These variations in immune cell composition may partially explain the differing efficacy of therapeutic strategies for ischemic stroke across age groups. Moreover, the inflammatory mediators identified in this study could represent potential molecular targets for novel therapeutic interventions, particularly those aimed at modulating the signaling pathways involved [9].

Our findings align with the concept of inflammaging [38]. Elderly patients exhibited higher basal expression of proinflammatory cytokines and oxidative stress markers, reduced NK/NKT cell activity, and a shift toward T-cell–dominated inflammation [39]. These features are consistent with immune remodeling in aging—where chronic cytokine exposure, microglial priming and immunosenescence dampen early innate responses (NK/NKT), yet prolong and intensify adaptive immune-mediated injury [40]. In contrast, young patients demonstrated rapid NK/NKT cell recruitment, efficient antioxidant response and earlier resolution of inflammation, reflecting a non-inflammatory immune profile.

Collectively, our findings suggest that the pathogenesis of ischemic stroke results from the interplay between neuronal necrosis, oxidative stress activation, and immune response, with age emerging as a critical factor determining the balance among these mechanisms and influencing clinical outcomes. Younger patients demonstrated more effective antioxidant defense, active NK cell infiltration, and initiation of a compensatory anti-inflammatory response. Moreover, the age-specific distribution of time to death—hyperacute in middle age versus delayed to Day 3 in the young—mirrors distinct immune and oxidative trajectories, supporting age-tailored therapeutic windows that target edema ultra-early and innate immune/redox pathways within 48–72 h, respectively. In contrast, elderly patients exhibited predominant lipid peroxidation, enhanced expression of proinflammatory cytokines, reduced NK cell activity, and a marked increase in T lymphocyte infiltration. The identified age-related differences in the morphological, biochemical, and immunological manifestations of ischemic stroke emphasize the need for the development of differentiated, age-specific therapeutic strategies that consider the dynamics of NK and T lymphocyte activation and migration, thereby minimizing secondary brain tissue damage.

## 4. Materials and Methods

**Figure 7:**
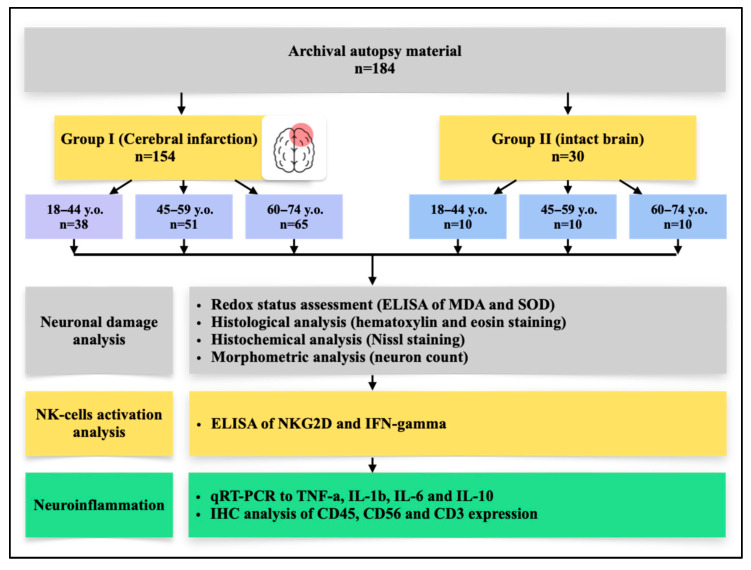
Study design.

For this retrospective descriptive study, archived autopsy material was obtained from 184 patients, who were subsequently stratified into study groups and age subgroups according to the experimental design (Figure 7).

### 4.1. Study Design

The control group (n = 30) consisted of autopsy specimens from patients whose deaths resulted from extracranial causes, with the brain considered conditionally intact. Causes of death in this group included traumatic injury to the brainstem, rupture of a cardiac or aortic aneurysm, rupture of esophageal varices, fatal cardiac arrhythmias, or electrocution, all without macro- or microscopic evidence of ischemic injury to the cortical structures of the brain at autopsy. Cases with systemic inflammatory complications were excluded. H&E examination confirmed absence of inflammatory cell infiltration in the cerebral cortex.

The IS (Ischemic stroke) group (n = 154) included frontal lobe cortical specimens from patients with a confirmed diagnosis of cerebral infarction (ICD-10 codes I63.3 and I63.4), verified through clinical and anamnestic data, neuroimaging (CT/MRI of the brain), and pathological examination. Only cases of ischemic stroke of cardioembolic or atherothrombotic origin (according to the TOAST classification) in the territory of the anterior or middle cerebral arteries were selected. In all cases, the causes of death were brain edema with displacement syndrome, acute cardiovascular failure, pulmonary embolism, and/or gastrointestinal bleeding. For analysis, brain tissue fragments were collected from patients who died within one week (acute phase) after the presumed or established onset of the disease.

Exclusion criteria for both groups included: hemorrhagic stroke, infarctions without involvement of the frontal cortex, cryptogenic and mixed stroke subtypes according to the TOAST classification, and the absence of clinicopathological verification of ischemic stroke for Group I. In addition, cases with significant concomitant pathology of the central nervous system (including neurodegenerative diseases, neoplasms, or traumatic brain injury), systemic hematologic, autoimmune, or oncological disorders, and acute bacterial and/or viral infections affecting the brain (including HIV, viral hepatitis B and C, syphilis, and sepsis) were excluded. Patients with documented alcohol or drug dependence were also excluded. Furthermore, cases with unsatisfactory autopsy material quality (autolysis, insufficient tissue volume, or technical errors during sampling, fixation, and/or storage) were not included in the analysis.

All brain samples were collected within six hours postmortem and processed into paraffin-embedded tissue blocks following standard histological protocols. Informed consent for pathological examination and use of tissue for research purposes was obtained from the patients’ next of kin. The study was approved by the Local Ethics Committee of Sechenov University (Protocol No. 13-25, dated 5 June 2025).

### 4.2. Enzyme-Linked Immunosorbent Assay (ELISA)

Fragments of the frontal lobe cerebral cortex were homogenized and centrifuged for 5 min at 1000 rpm on an ice bath. The resulting 10% homogenate was then centrifuged again, and the supernatant was collected for further analysis. To determine the levels of malondialdehyde (MDA), superoxide dismutase (SOD), the NK cell receptor NKG2D, and interferon-gamma (IFN-γ), commercial ELISA kits were used according to the manufacturers’ protocols: Malondialdehyde ELISA Kit (ab287797, Abcam, Waltham, MA, USA), Human Superoxide Dismutase 1 ELISA Kit (ab309312, Abcam, Waltham, MA, USA), Human NKG2D ELISA Kit (A303099, Antibodies.com LLC, St. Louis, MO, USA), and Human Interferon Gamma ELISA Kit (ab300323, Abcam, Waltham, MA, USA). Concentrations were calculated from standard curves using linear regression analysis and expressed in ng/mL for MDA and SOD, and pg/mL for NKG2D and IFN-γ.

### 4.3. Histological Examination

For histological analysis, 2-μm-thick sections were cut from paraffin-embedded tissue blocks, deparaffinized, and dehydrated. The sections were then stained with hematoxylin and eosin (H&E) following standard procedures. The resulting slides were examined under a Leica DM2000 light microscope (Leica Microsystems, Wetzlar, Germany).

### 4.4. Histochemical Examination

Nissl staining was performed to assess neuronal morphology and distribution in the cerebral cortex. Paraffin sections were deparaffinized and rehydrated through a descending ethanol series to distilled water, followed by staining with an aqueous cresyl violet solution for 5–10 min at room temperature. After staining, sections were differentiated in 96% ethanol to achieve optimal contrast, rinsed in distilled water, dehydrated through ascending ethanol concentrations, cleared in xylene, and mounted in Canadian balsam. Neuronal counts were performed under a light microscope at ×400 magnification in at least five randomly selected fields per sample.

### 4.5. Multiplex Immunohistochemical Analysis

Multiplex immunofluorescence staining was performed using the Opal™ 7-Color Fluorescence Immunohistochemistry Kit (Akoya Biosciences, Marlborough, MA, USA) according to the manufacturer’s protocol. After deparaffinization and rehydration, antigen retrieval was carried out in Tris-EDTA buffer (pH 9.0) using a microwave oven for 15 min, followed by blocking of endogenous peroxidase activity.

*First staining round.* Sections were incubated for 60 min with the primary antibody against CD45 (Clone HI30, Thermo Fisher Scientific, Waltham, MA, USA; 1:500). After washing, slides were treated with the HRP Ms + Rb polymer for 10 min, followed by incubation with the Opal 647 fluorophore for 10 min. Bound primary and secondary antibodies were then removed by repeated microwave treatment in Tris-EDTA buffer (pH 9.0) as described above.

*Subsequent staining rounds.* The procedure was repeated with antibodies against CD56 (Clone 56C04, Thermo Fisher Scientific, Waltham, MA, USA; 1:500) and CD3 (Clone OKT3, Thermo Fisher Scientific, Waltham, MA, USA; 1:500). After incubation with the HRP Ms + Rb polymer for 10 min, the slides were treated with Opal 488 and Opal 540 fluorophores, each for 10 min. Nuclei were counterstained with DAPI for 5 min, and the sections were mounted using ProLong™ Diamond Antifade Mountant (Thermo Fisher Scientific, Waltham, MA, USA).

*Visualization and quantitative analysis.* Multispectral images were acquired using the Vectra^®^ Polaris Automated Quantitative Pathology Imaging System (Akoya Biosciences, Marlborough, MA, USA) at 20× magnification. Whole-slide images were processed and analyzed using inForm^®^ Image Analysis Software v2.6 (Akoya Biosciences, Marlborough, MA, USA).

For each case, the numbers of NK cells (CD45^+^CD56^+^CD3^−^ phenotype), NKT cells (CD45^+^CD56^+^CD3^+^ phenotype), and T lymphocytes (CD45^+^CD56^−^CD3^+^ phenotype) were quantified in five randomly selected high-power fields (×400). The mean number of cells per 1 mm^2^ ± standard deviation (SD) was calculated. Additionally, the ratio of CD45^+^CD56^+^CD3^+^/CD45^+^CD56^+^CD3^−^ cells was determined to assess the proportion of activated NK T cells to the total NK cell population.

### 4.6. Quantitative Real-Time PCR (qRT-PCR) Analysis

The expression levels of mRNA for proinflammatory cytokines (IL-1β, IL-6, TNF-α) and the anti-inflammatory cytokine IL-10 were evaluated using quantitative real-time PCR (qRT-PCR). Tissue samples were homogenized according to standard protocols. Total RNA was extracted using the RNeasy Plus Mini Kit (QIAGEN GmbH, Hilden, Germany, The Netherlands). Complementary DNA (cDNA) synthesis was performed using the SuperScript™ VILO™ Master Mix (Invitrogen, Waltham, MA, USA).

The obtained cDNA samples were subjected to qRT-PCR using the ABsolute™ Blue QPCR Mix (Thermo Scientific, Waltham, MA, USA) with SYBR Green I detection dye. Amplification was carried out on the StepOne™ Real-Time PCR System (Applied Biosystems, Waltham, MA, USA) following the manufacturer’s instructions. Gene expression analysis was performed using the comparative threshold cycle (ΔCt) method, and relative expression levels were calculated according to the established protocol.

β-actin was used as a housekeeping reference gene for normalization. Primer sequences were designed based on publicly available DNA and mRNA data from the NCBI database using the Primer-BLAST tool (https://www.ncbi.nlm.nih.gov/tools/primer-blast/, accessed on 19 September 2025) (Table 2).

### 4.7. Statistical Analysis

Quantitative data were processed using SPSS Statistics 12 for Windows (IBM Analytics, Armonk, NY, USA). Continuous variables were analyzed using one-way ANOVA with Tukey’s or Bonferroni-adjusted post hoc tests when normally distributed, and the Kruskal–Wallis test with Dunn’s post hoc correction when non-normally distributed. Categorical variables were compared using the Pearson χ^2^ test, or Fisher’s exact test when expected cell counts were <5. Normality was assessed using the Shapiro–Wilk test. Pairwise comparisons between groups in the morphological analysis were performed using the Mann–Whitney U test with Bonferroni correction. Data for Days 4–7 were included mainly for descriptive purposes due to the small number of cases. Statistical comparisons were only performed when assumptions were met; in other cases, data were reported without inferential testing. The results are presented as mean ± standard deviation (SD) and, where applicable, with a 95% confidence interval (CI) for clinical data. A *p*-value ≤ 0.05 was considered statistically significant.

## 5. Conclusions

This study provides a comprehensive demonstration of age-related characteristics of neuroinflammation in ischemic stroke using human autopsy material. It was shown that in young patients, early post-stroke infiltration of the penumbra is accompanied by active migration of NK and NKT cells, as evidenced by increased expression of the NKG2D receptor. These cells exhibit high cytotoxic and proinflammatory activity, as indicated by elevated levels of IFN-γ, TNF-α, IL-1β, and IL-6. In contrast, elderly patients display dysregulation of the inflammatory response and a predominance of T lymphocytes within the infiltrate, along with reduced cytotoxic activity of NK cells and a more pronounced proinflammatory cytokine profile. The findings reveal novel aspects of the interaction between innate and adaptive immunity in the pathogenesis of ischemic stroke and highlight the importance of considering patient age when developing therapeutic strategies aimed at suppressing neuroinflammation and protecting neurons from secondary damage in the penumbra. Thus, our data suggest that age-related neuroinflammation after stroke may be interpreted through the lens of inflammaging, where chronic baseline inflammation and immunosenescence modulate acute innate and adaptive immune responses.

## Figures and Tables

**Table 1:** Clinical distribution of patients with IS in different age groups.

Parameter	M ± SD (95% CI)/n (%)			*p*
	Young (n = 38)	Middle (n = 51)	Elderly (n = 65)	
Age and gender composition of patients				
age, years	34.8 ± 4.2 (33.42–36.18)	51.1 ± 4.8 (49.75–52.45)	69.3 ± 5.4 (67.96–70.64)	**<0.0001 ^1^**
male	30 (78.9%)	34 (66.7%)	47 (72.3%)	0.315 ^2^
female	8 (21.1%)	17 (33.3%)	18 (27.7%)	
Diagnosis				
ICD-10:				
I63.3	15 (39.5%)	28 (54.9%)	36 (55.4%)	0.164 ^2^
I63.4	23 (60.5%)	23 (45.1%)	29 (44.6%)	0.173 ^2^
I67.8	1 (2.6%)	13 (25.5%)	**57 (87.7%)**	**<0.0001 ^2^**
SSS-TOAST:				
atherosclerosis	15 (39.5%)	28 (54.9%)	36 (55.4%)	0.164 ^2^
cardioembolism	23 (60.5%)	23 (45.1%)	29 (44.6%)	0.173 ^2^
Risk factors				
smoking	21 (55.3%)	29 (56.9%)	38 (58.5%)	0.95
arterial hypertension	34 (89.5%)	48 (94.1%)	64 (98.5%)	0.135
hypodynamia	9 (11.6%)	17 (33.3%)	**55 (84.6%)**	**<0.0001 ^2^**
obesity	18 (47.3%)	29 (56.9%)	28 (43.1%)	0.140
hyperlipidemia	22 (57.9%)	41 (80.4%)	**63 (95.4%)**	**<0.0001 ^2^**
Time of death				
Day 1	6 (15.8%)	**24 (47.0%)**	26 (40.0%)	**0.0073 ^2^**
Day 2	9 (23.7%)	12 (23.5%)	13 (20.0%)	0.8682
Day 3	**13 (34.2%)**	6 (11.8%)	13 (20.0%)	**0.035 ^2^**
Day 4	5 (13.2%)	2 (3.9%)	3 (4.6%)	0.1562
Day 5	2 (5.3%)	3 (5.9%)	6 (9.3%)	0.6867
Day 6	2 (5.3%)	1 (2.0%)	3 (4.6%)	0.6738
Day 7	1 (2.5%)	3 (5.9%)	1 (1.5%)	0.4112

For comparisons between groups, the following were used: ^1^—one-way ANOVA with post hoc; ^2^—Pearson’s χ^2^-test; statistically significant differences are shown in bold, *p* ≤ 0.05.

**Table 2:** Forward and reverse primers used for qRT-PCR analysis of mRNA expression of target genes (human-specific sequences).

Gene	Forward Primer (5′ → 3′)	Reverse Primer (5′ → 3′)
*IL-1β*	AGCTACGAATCTCCGACCAC	CGTTATCCCATGTGTCGAAGAA
*IL6*	ACTCACCTCTTCAGAACGAATTG	CCATCTTTGGAAGGTTCAGGTTG
*TNFα*	CCTCTCTCTAATCAGCCCTCTG	GAGGACCTGGGAGTAGATGAG
*IL-10*	TCTCCGAGATGCCTTCAGCAGA	TCAGACAAGGCTTGGCAACCCA
*β actin*	CATGTACGTTGCTATCCAGGC	CTCCTTAATGTCACGCACGAT

## Data Availability

The original contributions presented in this study are included in the article. Further inquiries can be directed to the corresponding author.
